# Correlation of Serum Lipoprotein Ratios with Insulin Resistance in Infertile Women with Polycystic Ovarian Syndrome: A Case Control Study 

**DOI:** 10.22074/ijfs.2016.4765

**Published:** 2016-04-05

**Authors:** Aisa Ghaffarzad, Reza Amani, Mahzad Mehrzad Sadaghiani M.D.3, Masoud Darabi, Bahman Cheraghian

**Affiliations:** 1 Department of Nutrition, School of Paramedicine, Nutrition and Metabolic Diseases Research Center, Ahvaz Jundishapur University of Medical Sciences, Ahvaz, Iran; 2 Department of Nutrition, University Health Research Institute, Diabetes Research Center, Ahvaz Jundishapur University of Medical Sciences, Ahvaz, Iran; 3 Women’s Reproductive Health Research Centre, Department of Infertility and Reproductive, Tabriz University of Medical Sciences, Tabriz, Iran; 4 Laboratory of Chromatography, Department of Biochemistry and Clinical Laboratories, School of Medicine, Tabriz University of Medical Sciences, Tabriz, Iran; 5 Department of Epidemiology and Biostatistics, Ahvaz Jundishapur University of Medical Science, Ahvaz, Iran

**Keywords:** Lipoprotein, Infertility, PCOS, Insulin Resistance

## Abstract

**Background:**

Dyslipidemia and insulin resistance (IR), occurring in most infertile women with polycystic ovarian syndrome (PCOS), increase the risk of cardiovascular disease (CVD) and type 2 diabetes. This study aimed to assess the relationships between lipoprotein ratios and IR in PCOS women.

**Materials and Methods:**

Thirty six infertile women with PCOS selected based on Androgen Excess Society (AES) criteria and 29 healthy women matched for age were recruited to this case-control study. After physical measurements, fasting serum glucose (Glu), insulin and lipid profile levels [triglycerides (TGs), total cholesterol (TC), low-density lipoproteincholesterol (LDL-C) and high-density lipoprotein-cholesterol (HDL-C)] were measured, while lipoprotein ratios (TC/HDL-C, LDL-C/HDL-C, TG/HDL-C) were calculated. IR was also calculated using homeostasis model assessment (HOMA)-IR. The optimal cutoffs of lipoprotein ratios in relation to HOMA-IR were calculated based on the Receiver Operating Characteristics (ROC) curve analysis using the area under curve (AUC).

**Results:**

Waist circumference (WC), insulin levels, HOMA-IR, TG levels, and all lipoprotein ratios were significantly higher, while HDL-C was lower in PCOS group as compared to healthy controls. All lipoprotein ratios, TG levels, and WC are significantly correlated with insulin levels and HOMA-IR. Among lipoprotein ratios, the highest AUC of the ROC belonged to TG/HDL-C ratio with sensitivity of 63.6% and specificity of 84.4% (TG/HDL-C>3.19) as a marker of IR in infertile PCOS women.

**Conclusion:**

Lipoprotein ratios, particularly TG/HDL-C, are directly correlated with insulin levels and can be used as a marker of IR (HOMA-IR) in infertile PCOS patients.

## Introduction

Polycystic ovary syndrome (PCOS) is the most common gynecological endocrinopathy disorder and the frequent cause of oligo-ovulatory infertility (1). Abnormalities with ovulation are the cause of infertility in about one third of couples attending infertility clinics that count for 90% of these cases (2). PCOS is generally characterized by chronic anovulation, hyperandrogenism and ovarian polycystic changes that are detected by an ultrasound scan in a clinic (3). The estimated prevalence of PCOS based on the criteria used for diagnosis and recruitment process of the study population has been reported between 2.2 and 26% in different countries (4). Although the etiology of PCOS is still unknown, it has been demonstrated that PCOS is a metabolic disorders rather than a reproductive endocrine disease (3). Insulin is a key component in the pathophysiology of PCOS (1). On average, PCOS patients have higher triglyceride (TG), lower high density lipoprotein-cholesterol (HDL-C) and higher low density lipoprotein-cholesterol (LDL-C) levels than their non-PCOS matched group (5). Insulin resistance (IR), hyperinsulinaemia and dyslipidemia are diagnosed among 50 to 70% of patients with PCOS (6). There is a drastic improvement in PCOS complication when is accompanied by modulation of IR (1). Therefore, PCOS is associated with increased risk of metabolic abnormalities, indicating that the patients are at the risk of developing type 2 diabetes and cardiovascular disease (CVD) (3).

Despite modern treatment options for infertilities and considering economic aspects, it is reasonable to give specific attention to cost effective and easily applied methods for predicting metabolic abnormalities at population level (7). Routine methods for measuring IR are hyperinsulinemiceuglycemic clamp technique (a gold standard to assess insulin sensitivity) (8), homeostasis model assessment (HOMA)-IR, Bennett index, Li Guangwei index, quantitative insulin sensitivity check index (QUICKI), and fasting serum glucose (Glu)/insulin ratio (G/I). Due to being complex, expensive and time-consuming, the latter methods are of limited use in clinical and epidemiological studies (9). Thus for daily clinical practice, it is necessary to use other methods for measuring IR, which are lower in costs and applicable to the general population.

In order to provide a new idea to evaluate IR in infertilities associated with PCOS, the possibility of establishing the values of total cholesterol (TC)/ HDL-C, TG/HDL-C, and LDL-C/HDL-C ratios, waist circumference (WC) as surrogates, as well as LDL-C, TC, and TG levels to estimate insulin levels and IR was investigated. By using Receiver Operating Characteristic (ROC) curves in our subjects, the accuracy of the mentioned parameters was recived.

## Materials and Methods

### Subjects

In this case-control study, subjects were selected among women aged 19 to 35 years who visited a private reproductive medical center, Tabriz, Iran, during the period of February till April 2013, for infertility due to PCOS. Selection was done by the standardized protocol for the initial evaluation. A total of 35 patients were identified as PCOS cases according to the Androgen Excess Society (AES, 2006) criteria (1), while 29 age-matched healthy women (without any infertility and PCOS disorders) were recruited in the study as the control group. Inclusion criteria for case group were as follows: married, clinical and/or biochemical hyperandrogenism, and ovarian dysfunction (oligoanovulation and/or polycystic ovaries detected by ultrasound scans). Exclusion criteria were as follows: congenital adrenal hyperplasia, androgensecreting tumors, taking androgenic/anabolic medications, Cushing syndrome, severe IR syndrome, thyroid dysfunction, hyperprolactinemia, diabetes, hypertension, CVD, taking vitamins and supplements during the 3 months prior to the study, evidence of recent or recurrent infection, and smoking or drinking alcohol.

### Physical measurements

Body weight was measured without shoes with minimal amount of clothing using a digital scale (SECA, Germany) to the nearest 0.1 kg. Height was measured using a non-stretchable stadiometer (SECA, Germany) to the nearest 0.1 cm. Body mass index (BMI) was calculated as weight in kg divided to height in squared meters. WC was measured at the midpoint between the lowest rib and the top of the lateral border of iliac crest during minimal respiration. Systolic blood pressure (SBP) and diastolic blood pressure (DBP) were measured using Spot Vital Signs Device (Welch Allyn, USA). Participants were asked to lie down and relax for approximately 8 to 10 minutes, after which three blood pressure measurements were recorded at fiveminute intervals.

### Blood analysis

After 12-hour overnight fast, blood samples were collected. Serum and plasma samples were separated using a centrifuge (Beckman Coulter Inc., USA) at 1500 rpm for 15 minutes. Fasting insulin levels were measured using enzyme-linked immunosorbent assay (ELISA) kits (Monobind Inc., USA). Fasting plasma Glu was measured using enzymatic procedures by an automatic analyzer (Abbott, USA). IR was estimated by HOMA using the following formula: HOMA- IR=fasting insulin (μU/ml)×fasting Glu (mg/dl)/405 (10). The concentrations of TC and TG were measured using enzymatic procedure with commercial kits (Pars Azmon, IRI), while HDL-C was measured by a direct method using polyethylene-glycol-pretreated enzymes by an automatic analyzer (Abbott, USA). LDL-C was calculated using Friedewald’s formula (11). Lipoprotein ratios (TC/ HDL-C, TG/HDL-C and LDL-C/HDL) were then calculated.

### Ethical considerations

This study was approved by the Medical Ethics Committee of Ahvaz Jundishapur University and all participants gave an informed consent before commencing the study. The code of Ethics Committee is ETH-702, and registered code of study is NRC-9110.

### Statistical analysis

Results were expressed as mean ± SD. Levene’s test for equality of variances was used. The differences between concerning continuous and categorical variables were analyzed using unpaired t test (or Mann-Whitney U test for non-normally distributed data) and λ^2^ test, respectively. Correlations were determined by Spearman correlation coefficient method. ROC curves were used to estimate the sensitivity and specificity of serum lipoprotein ratios to diagnose IR. P values less than 0.05 were considered statistically significant. All statistical analyses were performed using Statistical Package for Social Sciences 20.0 (SPSS, SPCC Inc., USA) software.

## Results

The control group was matched with the patient group for age. Although the values of BMI, BP, TC, LDL-C, TG and fasting serum Glu were found to be higher in the infertile PCOS group than in the control group, indicating that these differences were not statistically significant. A higher insulin level and HOMA-IR value were observed in patients group compared to the control group (P<0.001 and P=0.024, respectively). TG levels (P=0.009) as well as the values of TC/ HDL (P=0.002), TG/HDL (P=0.047), LDL/HDL (P=0.002) and WC (P<0.001) were significantly higher, while HDL-C levels (P=0.003) were lower in the cases compared to those of their healthy counterparts. The results are shown in Table 1.

HOMA-IR value in the patients showed a positive correlation with TG levels (r=0.56, P<0.01) as well as the values of TC/HDL-C (r=0.34, P<0.05), TG/HDL-C (r=0.49, P<0.01), LDL-C/HDL-C (r=0.33, P<0.05), and WC (r=0.37, P<0.05). However, HOMA-IR value showed no significant correlation with TC, LDL and HDL concentrations. Serum insulin levels are positively correlated with TG level (r=0.46, P<0.01), TC level (r=0.33, P<0.05), and TG/HDL value (r=0.39, P<0.05). We found no significant correlation between serum insulin levels and LDL-C, HDL-C, TC/HDL, LDL/ HDL and WC values in our patients. The results are shown in Table 2.

**Fig 1:**
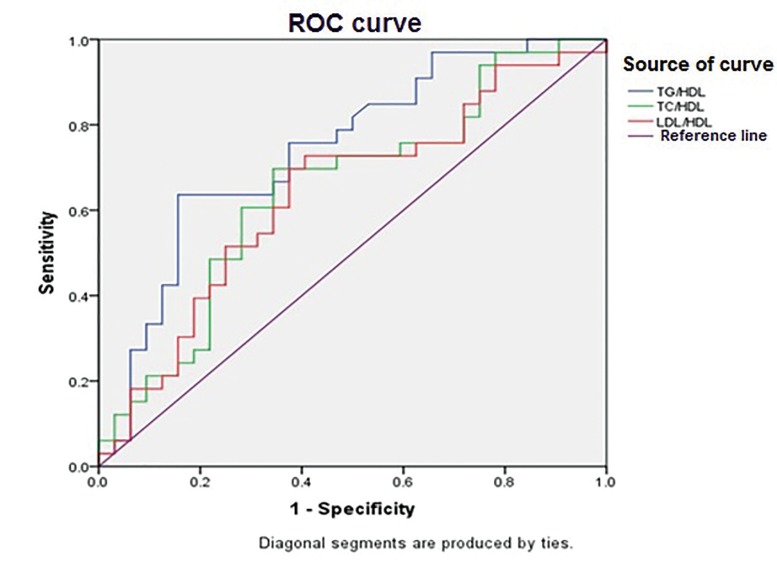
ROC curves of serum lipoprotein ratios for detection of insulin resistance in PCOS cases. Diagonal segments are produced by ties. PCOS; Polycystic overian syndrome and ROC; Receiver operating characteristic.

According to the ROC curve analysis, all lipid ratios (TG/HDL-C, TC/HDL-C, and LDL/HDLC) showed an area under curve (AUC) greater than 0.5. Thus, as an effective diagnostic marker for IR in PCOS patients, the AUC of TG/HDL-C was the highest with sensitivity of 63.6% and specificity of 84.4% (TG/HDL-C>3.19). The results are shown in Table 3 and Figure 1.

**Table 1:** Baseline and clinical characteristics of two groups (age range 19-35 years)


Variables	Infertile PCOS (n=36)	Healthy control (n=29)	P valuea^a^

Age	26.36 ± 4.2	27.96 ± 2.47	0.107
BMI (kg/m2)	26.72 ± 4.39	25.55 ± 4.3	0.286
BMI (%)c BMI≥25	72.2	48.3	0.049
WC (cm)	94.77 ± 10.36	85.06 ± 8.48	<0.001
SBP (mmHg)	118.66 ± 8.98	116.89 ± 6.03	0.209
DBP (mmHg)	78.19 ± 6.98	76.37 ± 5.15	0.274
Fasting serum Glu (mg/dL)	94.47 ± 11.88	89.86 ± 8.25	0.081
Insulin (μU/mL)b	21.41 ± 14.14	16.24 ± 11.55	0.029
HOMA-IRb	5.16 ± 3.72	3.41 ± 2.53	0.024
TC (mg/dL)	214.83 ± 43.97	202.68 ± 46.44	0.285
TG (mg/dL)	139.28 ± 66.98	98.17 ± 50.72	0.009
HDL-C (mg/dL)	42.88 ± 10.2	52.06 ± 13.71	0.003
LDL-C (mg/dL)	143.69 ± 36.25	129.51 ± 35.70	0.119
TC/HDL-C ratio	5.16 ± 1.22	4.11 ± 1.36	0.002
TG/HDL-C ratio	3.62 ± 2.17	2.44 ± 2.52	0.047
LDL-C/HDL-C ratio	3.44 ± 0.98	2.62 ± 1.02	0.002


PCOS; Polycystic ovarian syndrome, BMI; Body mass index, WC; Waist circumference, SBP; Systolic blood pressure, DBP; Diastolic blood pressure, Glu; Glucose, HOMA-IR; Homeostasis model assessment of insulin resistance, TC; Total cholesterol, TG; Triglyceride, HDL-C; High density lipoprotein-cholesterol, LDL-C; Low density lipoprotein-cholesterol, ^a^; Statistical analyses performed by unpaired t test for comparison, ^b^; Statistical analyses performed by Mann-Whitney U test and ^c^; Statistical analyses performed by Chi-squared test. Data are the mean ± SD.

**Table 2:** Spearmanʼs correlations of lipid profile, lipoprotein ratios and WC values with serum insulin level and IR in infertile women with PCOS


Variables	Serum insulin levels	IR

TGs	0.46^b^	0.56^b^
TC	0.33^a^	0.316
LDL-C	0.29	0.28
HDL-C	0.14	0.08
TC/HDL	0.3	0.34^a^
TG/HDL	0.39^a^	0.49^b^
LDL/HDL	0.28	0.33^a^
WC	0.32	0.37a


IR; Insulin resistance, PCOS; Polycystic ovary syndrome, WC; Waist circumference, TC; Total cholesterol, TG; Triglyceride, HDL-C; High density lipoprotein-cholesterol, LDL-C; Low density lipoprotein-cholesterol, ^a^; P<0.05 and ^b^; P<0.01.

**Table 3:** Serum lipoprotein ratios, AUC, cut-off points and sensitivity and specificity calculated from ROC curves for the detection of PCOS with IR


Serum lipoprotein ratios	AUC± SE	95% CI	Cut-off point	Infertile PCOS patients		P value
				Sensitivity (%)	Specificity (%)	

TG/HDL	0.743 ± 0.062	0.622-0.864	3.19	63.6	84.4	0.001
TC/HDL	0.651 ± 0.069	0.515-0.786	4.37	69.7	65.6	0.037
LDL/HDL	0.638 ± 0.070	0.502-0.775	2.84	69.7	63.5	0.055


CI; Confidence interval, IR; Insulin resistance, AUC; Area under curve area, ROC; Receiver operating characteristic, PCOS; Polycystic ovarian syndrome, TC; Total cholesterol, TG; Triglyceride, HDL; High density lipoprotein and LDL; Low density lipoprotein.

## Discussion

Among the factors responsible for a reduction in fecundity and successful pregnancy, the hormonal changes associated with various factors are considered as an important cause for interrupting normal ovulatory menstrual cycles (12). Among these factors, visceral adiposity is a common finding in PCOS patient, even when the subjects are not classified as overweight (25<BMI<29.9) (13). According to our findings, we observed a significant different in WC between groups. Although the difference in BMI index between cases and controls was not significant, but in case group, percentage of overweight BMI was higher than that of controls. Also there was a significantly positive correlation between WC and IR. Our Findings are in agreement with those of some previous studies (13-15). However, the latter results differ from those of the study conducted by Iuhas et al. (16). In their study, visceral fat area showed no significant difference in PCOS and healthy subjects, which might be due to the difference in method of measuring visceral fat and larger sample size. Pathophysiology of PCOS is unknown. It is regarded as an endocrinal disorder due to IR, which presents in about 70% of PCOS patients (17, 18). In PCOS patients, IR is mostly associated with dyslipidemia. Methods used for measuring IR are mostly sophisticated and expensive that are not applicable for epidemiological studies. Hence, more reasonable methods for IR measurements have been investigated in several studies, of which lipoprotein ratios were proposed for the identification of IR as an alternative method. Our investigation was carried out in order to provide evidences for the application of lipoprotein ratios as an indicator of IR in infertile PCOS women. In this investigation, PCOS was diagnosed by AES criteria, while for the first time, subjects were selected among infertile PCOS women.

This study showed the case group had higher TG levels and lower HDL-C levels compared to control group. While no significant difference was detected in TC and LDL-C levels between groups. Most studies have shown low levels of HDL-C in women with PCOS, but composition of HDL in PCOS is still unknown. There is still a need for further studies in order to determination of the HDL-C composition in these patients. One of the mechanisms that could explain the observed difference is the activity of hepatic lipase (HL) enzyme induced by IR and hyperandrogenemia, which removes lipid from HDL and plays as a key role of the lipid-depleted HDL particles in PCOS patients. Also insulin-resistant states along with low HDL levels are frequently associated with hypertriglyceridemia. However, another possible mechanism of dyslipidemia in PCOS could be a reduction in clearance of triglyceride-rich proteins (19).

Result of this study demonstrated a significant association in TC/HDL-C, TG/HDL-C and LDLC/ HDL-C ratios and TG with IR (HOMA-IR) in PCOS patients. In a study on women with PCOS, Xiang et al. (20) also suggested that serum lipoprotein ratios could be used as a marker of IR due to the significant positive correlation of the indices with IR. However, in their study, Rotterdam criteria were used for diagnosing PCOS, so there was a significant difference in terms of BMI between case and control groups, which could be a confounding factor. Hence the present study was designed more specifically by use of updated criteria (AES) on infertile women for diagnosing PCOS. Moreover in our study BMI was not significantly different between case and control groups, which could justify the confounding impact of BMI on results. Serum lipoprotein ratios were also reported to be significantly correlated to IR in type 2 diabetes patients (21). Furthermore TG/HDL could be considered as a simple reliable indicator to determine IR in healthy (22) and severely obese nondiabetic individuals (23). Our results on women with ovulatory disorder infertility also confirmed these findings. ROC curve analysis showed that TG/HDL-C, TC/HDL-C, and LDL-C/HDL-C with an AUC greater than 0.5 were effective and useful diagnostic markers for IR in infertile PCOS women. AUC of TG/HDL-C was the highest with sensitivity of 63.6% and specificity of 84.4% (TG/ HDL-C>3.19). Xiang et al. (20) have also shown that AUC of TC/HDL-C had the highest sensitivity and specificity (TC/HDL-C>3.6). This discrepancy could be partially due to lesser sample size in our study or possible racial differences.

Future studies with higher sample size and more specific markers are needed to show the correlation between lipid ratios and IR in an extended level.

## Conclusion

Our investigation demonstrated that despite the routine methods used for measuring IR, TC/ HDL-C, TG/HDL-C, and LDL/HDL ratios could be regarded as simple, reliable and economic indicators of IR in PCOS infertile women. Moreover the combination of higher serum lipoprotein ratios and TG levels with abdominal obesity may predispose a group of patients to more marked risks for IR.
